# Trends in clinical and pharmacological profiles of severe asthma in the era of biologics in the Brazilian public health system: real-world evidence from a tertiary outpatient clinic

**DOI:** 10.36416/1806-3756/e20250106

**Published:** 2025-09-22

**Authors:** Carolina Fernanda Oliveira Santos Tallarico, Priscila Carla Moura Honório, Vanusa Barbosa Pinto, Alberto Cukier, Rodrigo Abensur Athanazio, Regina Maria de Carvalho-Pinto

**Affiliations:** 1. Divisao de Farmacia, Instituto Central, Hospital das Clínicas, Faculdade de Medicina, Universidade de Sao Paulo - HCFMUSP - Sao Paulo (SP) Brasil.; 2. Unidade de Vias Aéreas, Divisao de Pneumologia, Instituto do Coração - InCor - Hospital das Ciínicas, Faculdade de Medicina, Universidade de Sao Paulo - HCFMUSP - São Paulo (SP) Brasil.

## TO THE EDITOR:

*Protocolos Clínicos e Diretrizes Terapêuticas* (PCDT, Clinical Protocols and Therapeutic Guidelines) are documents that are issued by the Brazilian National Ministry of Health and that must be followed by *Sistema Único de Saúde* (SUS, Brazilian Unified Health Care System) managers. Before the PCDT for asthma were updated in 2021,^1^ patients with severe asthma treated in the SUS did not have access to biologics and experienced a high disease burden. In 2012, we published data describing the characteristics of our outpatient population with severe asthma, highlighting this significant disease burden.^2^

Omalizumab has been available for the treatment of severe asthma for more than a decade; however, in 2016 the Brazilian National Committee for Technology Incorporation decided against the provision of treatment with omalizumab within the scope of the SUS.^3^ In response, we at the Pulmonology and Allergy/Immunology Outpatient Clinic of the University of São Paulo School of Medicine *Hospital das Clínicas*, located in the city of São Paulo, Brazil, developed a protocol to enable patient access to omalizumab treatment.^4^ We also sought to develop researcher-initiated projects designed prior to the incorporation of biologics and presented our findings at a number of conferences, sharing our clinical experience.^5^ ^,^ ^6^

Biologics can reduce exacerbation rates, decrease oral corticosteroid (OCS) use, and improve asthma control. However, after treatment initiation, it is important to evaluate patient response within 6-12 months with the goal of reducing maintenance therapy, especially those associated with significant side effects. In this context, tapering OCS and high-dose inhaled corticosteroid (ICS) doses is particularly relevant.^7^ ^,^ ^8^

There are several ways to assess clinical response to biologic therapy, including a reduction in exacerbation frequency; improvement in symptom control and lung function; reduction in maintenance therapy; prevention of adverse effects; and enhanced patient satisfaction.^7^ ^,^ ^8^ When a satisfactory clinical response is achieved, it is recommended to reduce maintenance therapy to the lowest dose required to maintain disease control. In this context, strategies to minimize OCS use are especially important, given the potential for significant adverse effects.^7^

Since the 2021 update of the PCDT for asthma,^1^ patients with severe asthma treated in the SUS have had access to two biologics, namely, omalizumab and mepolizumab. Here, we present real-life outcomes in patients followed at our asthma outpatient clinic after the inclusion of omalizumab and mepolizumab in the SUS, as outlined in the updated PCDT for asthma. This was a retrospective observational study, the primary objective of which was to evaluate the clinical and pharmacological profiles of severe asthma patients treated at a tertiary outpatient clinic in the SUS. The study was approved by the local institutional review board (Protocol no. 7.058.172). 

The statistical analysis was descriptive in nature. Categorical variables were presented as absolute numbers and percentages. Numerical variables were expressed as mean ± standard deviation, depending on the distribution of the data. In the present study, handling of missing data was not applicable because data on the outcomes assessed (Asthma Control Test [ACT] scores, Asthma Control Questionnaire [ACQ] scores, exacerbations, and prescribed medications) are regularly collected through standardized procedures during each visit, and no data were missing. 

This was a convenience sample. Of a total of 60 severe asthma patients receiving omalizumab or mepolizumab for at least six months in accordance with the 2021 PCDT for asthma,^1^ 45 were included. Of those, 11 (18.3%) were excluded because of prior use of another biologic agent, and 4 (6.7%) declined to participate. After patients gave written informed consent, patient medical records and our electronic prescription system were retrospectively reviewed in order to collect data at baseline and at 24 and 48 weeks after initiation of biologic therapy, as well as to assess medical prescriptions. A total of 45 patients were evaluated at baseline and at 24 weeks, whereas 30 patients were evaluated at 48 weeks. This was due to the fact that 15 patients either discontinued therapy because of treatment failure or had an insufficient follow-up period. The data were entered into a Research Electronic Data Capture (REDCap; Vanderbilt University, Nashville, TN, USA) database. The use of other medications was ascertained from previous prescriptions, and short-acting β_2_ agonist (SABA) doses were recorded from our electronic prescribing system. Adherence to treatment with biologics was assessed on the basis of outpatient clinic attendance. 

Our patient population was predominantly female (77.8%) with uncontrolled asthma (mean ACT score, 14.5 ± 5.3; mean ACQ score, 2.5 ± 1.2). Most were using a high-dose ICS combined with a long-acting β_2_ agonist (LABA), except for one patient who was intolerant to LABAs. Approximately 67% had childhood onset asthma, and 55.6% were atopic. Patients experienced an average of 3.3 exacerbations in the preceding year and had impaired lung function (prebronchodilator percent predicted FEV_1_, 62 ± 22). The mean age at initiation of biologic therapy was 50 years, and 17.8% were former smokers. One third of the study participants were receiving maintenance OCS therapy, and 60% received mepolizumab. The mean number of comorbidities was 4.4. Adherence to biologic therapy was demonstrated by the fact that 71% and 80% of the study participants attended all scheduled appointments at weeks 24 and 48, respectively. 

We observed an improvement in asthma control following biologic therapy. At baseline, the mean ACT score was 14.5 ± 4.8 and the mean ACQ score was 2.5 ± 1.3, indicating uncontrolled asthma. At week 48, those scores improved to 17.8 ± 5.1 (a 3.3-point increase) and 1.5 ± 1.0 (a 1.0-point decrease), respectively (Table 1). Given that a change of 3 points in the ACT score and 0.5 points in the ACQ score is considered clinically significant,^1^ ^,^ ^8^ our results demonstrate a clinically significant improvement in asthma control. 

**Table 1:** Evaluation of severe asthma patients during follow-up.^a^

Variable	Baseline (n = 45)	Week 24 (n = 45)	Week 48 (n = 30)
No. of exacerbations	3 [0-11]	0 [0-5]	0 [0-4]
ACT score	14.5 ± 4.8	17.3 ± 5.5	17.8 ± 5.1
ACQ score	2.5 ± 1.3	1.7 ± 1.2	1.5 ± 1.0
ICS-LABA maintenance therapy	44 (97.8)	44 (97.8)	28 (93.3)
Mean daily dose of ICS + LABA, µg^b^	1,382 ± 341	1,350 ± 412	1,418 ± 409
ICS	26 (57.8)	23 (51.1)	17 (56.7)
Mean daily dose of ICS, µg^b^	2,227 ±1352	2,117 ± 1,388	2,276 ± 1,428
Total daily dose of ICS, µg^b^	1,696 ± 950	1,547 ± 775	1,742 ± 1,010
OCS	16 (35.5)	14 (31.1)	8 (17.8)
Total daily dose of OCS, mg^c^	21 ± 14	21 ± 14	17 ± 11
Rescue ICS + LABA	16 (35.6)	17 (37.8)	7 (23.3)
Mean daily dose of rescue ICS + LABA, µg	700	623	485
SABA	27 (60)	23 (51)	18 (60)
Mean daily dose of SABA, µg	625	543	522
Antibiotic therapy^d^	9 (20)	8 (17.8)	4 (13.3)
H1 antihistamines	19 (42.2)	19 (42.2)	14 (46.7)
Antileukotrienes	17 (37.8)	17 (62.2)	11 (36.7)
Proton pump inhibitor	39 (86.7)	38 (84.4)	25 (83.3)
Prokinetics	28 (62.2)	25 (55.6)	14 (46.7)
Nasal corticosteroid	34 (75.6)	35 (77.8)	23 (76.7)
Ipratropium bromide	17 (37.8)	19 (42.2)	9 (30)
Tiotropium bromide	11 (24.4)	7 (15.6)	8 (26.7)

ACT: Asthma Control Test; ACQ: Asthma Control Questionnaire; ICS: inhaled corticosteroid; OCS: oral corticosteroid; LABA: long-acting β_2_ agonist; and SABA: short-acting β_2_ agonist. ^a^Data presented as n (%), mean ± SD, or median [IQR]. ^b^ICS dose equivalent to budesonide. ^c^OCS dose refers to prednisone or equivalent. ^d^Azithromycin (3 times/week).

There have been few real-life studies evaluating the response to biologics in patients with severe asthma in Brazil. In a study evaluating severe asthma patients in a public hospital in the state of Paraná,^9^ there was an ACT score improvement of 4.8 points, which is comparable to the results observed in our study. In addition, the frequency of exacerbations decreased from an average of 3 per year to 0 per year, despite a reduction in the proportion of patients using OCSs (Table 1). 

**Figure 1:**
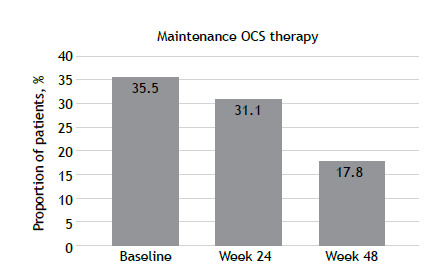
Proportion of patients receiving maintenance oral corticosteroid (OCS) therapy during follow-up.

At baseline, approximately 35% of patients were receiving maintenance OCS therapy. As can be seen in Table 1 and Figure 1, there was a relative reduction of approximately 49% in the proportion of patients using OCSs by week 48, decreasing from 35.5% to 17.8%. 

No significant changes were observed in the use of maintenance ICS-LABA therapy during follow-up, and more than 50% of the patients remained on additional ICS therapy alongside ICS + LABA throughout the evaluation period (Table 1). The lack of evidence supporting ICS step-down approaches in patients with severe asthma likely explains the more conservative approach to this intervention. The first evidence for ICS reduction in this context emerged in early 2024 with the use of benralizumab.^10^ Given that our patients were evaluated by the same medical team prior to each biologic dose, we can infer that adjustments to maintenance ICS-LABA therapy, OCSs, and other medications were tailored to each patient, even with the constraints of real-life clinical practice. 

To assess the use of therapy (ICS-LABA therapy or SABA therapy), we considered the total daily dose because it was not feasible to estimate the actual individual dose used, given that the evaluation was based on electronic prescription data. A reduction was observed in the proportion of patients who were prescribed ICS-LABA therapy for relief and in the mean ICS dose within this combination, decreasing from 700 µg at baseline to 485 µg at week 48. Similarly, the mean SABA dose decreased throughout the study period. (Table 1) 

Each of the study participants used an average of eight medications, some of which were prescribed for comorbidities related to asthma. It is important to consider the potential impact of those comorbidities, as well as other factors, on treatment response. However, because data were collected exclusively from our electronic prescribing system, we were unable to capture information on treatments outside our facility. 

Our study has some limitations. First, it was a single-center study conducted at a tertiary university hospital; therefore, caution is recommended when generalizing the data. Second, the small sample size and the descriptive and retrospective design of the study may also be subject to information gaps. Nonetheless, this was a real-life study conducted in a complex clinical setting and evaluating severe asthma patients treated by the same medical team while receiving biologic therapy. This approach reflects a personalized, patient-centered model of care, underscoring the importance of appropriate patient selection and close follow-up when prescribing high-cost treatments, especially in the context of limited financial resources. 

Our data describe the clinical and pharmacological profiles of severe asthma patients treated with biologics at a tertiary outpatient clinic in the Brazilian public health system. This real-life study demonstrates significant improvements in clinical control and exacerbation rates, despite a reduction in the proportion of patients using OCSs. However, our findings also suggest that a longer follow-up period may be required to reduce maintenance therapy further. The presence of comorbidities often leads to polypharmacy and increases the risk of self-medication, potentially complicating disease control. Our results underscore the importance of pharmacist involvement in patient care to identify treatment-related problems, optimize therapeutic management, and prevent inappropriate or irrational medication use. 
